# Pulmonary manifestations in a pediatric patient with ulcerative colitis: a case report

**DOI:** 10.1186/1752-1947-2-59

**Published:** 2008-02-25

**Authors:** Ryan S Carvalho, Lindsay Wilson, Carmen Cuffari

**Affiliations:** 1 The Johns Hopkins University School of Medicine, Department of Pediatrics, Division of Pediatric Gastroenterology and Nutrition, Baltimore, Maryland, USA

## Abstract

**Introduction:**

Although respiratory involvement has been described in patients with IBD, well-defined interstitial lung disease has not been reported, especially among children with ulcerative colitis.

**Case presentation:**

Herein, we present a case of an adolescent female with ulcerative colitis and extra-intestinal complications involving the lungs that were effectively treated with anti-metabolite therapy.

**Conclusion:**

Children with UC may manifest either interstitial or large airway pulmonary involvement. All children with suspected lung involvement should be screened for tuberculosis prior to starting immunosuppressive therapy.

## Introduction

The most prevalent pulmonary manifestation in either Crohn's disease (CD) or ulcerative colitis (UC) is non-specific airway inflammation [1-3]. Untreated, patients are at risk for developing bronchiolitis obliterans with organizing pneumonia, tracheal stenosis and bronchiectasis [4,5]. Although necrobiotic lung nodules are less common, they represent an important pulmonary manifestation of interstitial lung disease in patients with IBD [6]. In Pediatrics, the pulmonary manifestations of IBD have been recognized only in children with CD [7-9]. Herein, we describe a pediatric patient with ulcerative colitis and pulmonary manifestations that were effectively treated with immunosuppressive therapy.

## Case presentation

**Figure 1:**
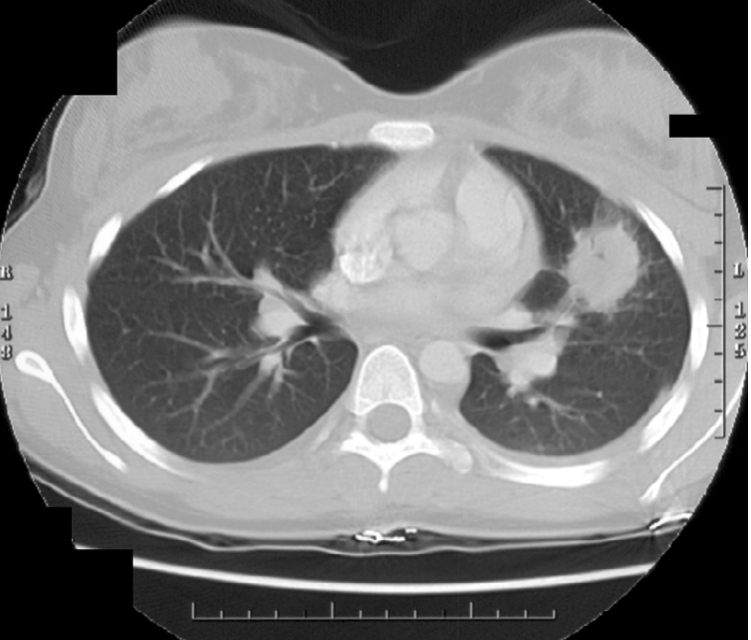
Chest CT scan showing a well-circumscribed homogeneous pulmonary mass (4 cm) within the lingual of the left lung in a newly diagnosed child with ulcerative colitis.

**Figure 2:**
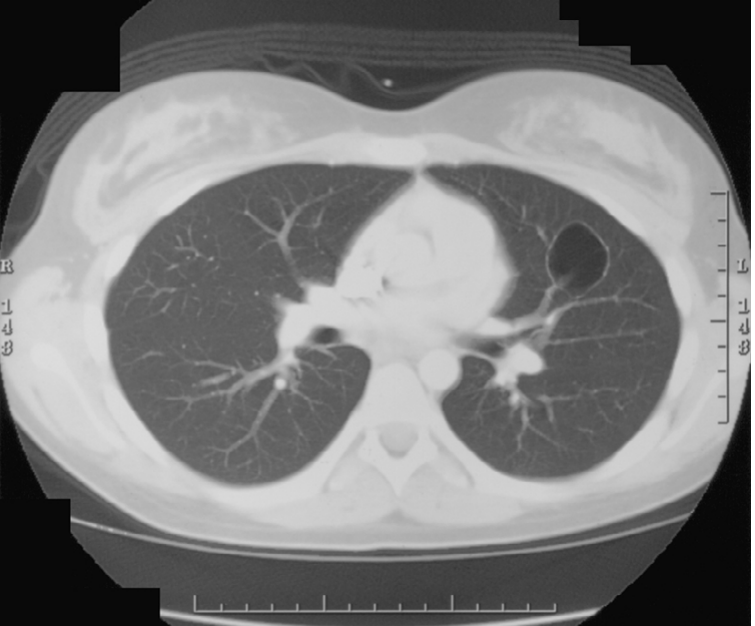
Follow-up (12 mo.) chest CT scan in the same patient on maintenance 6-mercaptopurine therapy with a residual cavitary lesion within the lingual of the left lung.

A 13.5 yr. old female with UC diagnosed a year prior, presented at a local hospital emergency room with a 1 month history of abdominal pain and diarrhea that progressed to frank hematochezia. Her symptoms were also associated with fever, night sweats, malaise, decreased appetite and weight loss. A chest and abdominal computerized tomography (CT) scan showed a 4 cm nodule in the lingula (Fig. 1a) and 2 smaller nodules in the left lower lobe. A CT-guided biopsy of the lingular mass showed no malignancy, but marked alveolar inflammation. The patient was also PPD negative, and all cultures, including blood, sputum and lung tissue for bacteria, atypical mycobacteria, virus and fungal organisms were negative.

The patient was transferred to The Johns Hopkins's Children's Center for further evaluation. Pulmonary functions testing showed mild obstructive, but no restrictive lung disease. A transbronchial biopsy of the lingular mass verified the presence of a necrobiotic nodule, and repeat tissue cultures were also negative. The patient continued to have frequent bloody diarrhea that was treated with intravenous corticosteroids, parenteral nutrition, antibiotics and mesalamine therapy. The patient's symptoms resolved within 5 days of initiating therapy. She was prescribed 6-mercaptopurine (1 mg/Kg/day) upon discharge from hospital, and has remained essentially asymptomatic up to 3 years in follow-up. A repeat chest CT done at 12 months post discharge showed complete resolution of the 2 small left lower lobe lesions, however, the lingular lesion was replaced with a residual thin-walled cyst measuring 2.4 × 3.4 cm in diameter (Fig. 1b).

## Discussion

Although extra intestinal manifestations are relatively common (13–45%) in patients with IBD [2], pulmonary manifestations are considered rare [1-3]. Moreover, while there have been reported cases of pulmonary manifestations in pediatric CD [7-9], this is the first reported case of interstitial lung involvement in a child with UC. In comparison, Camus and coworkers have described a number of pulmonary manifestations in adult patients with UC, including bronchiolitis obliterans with organizing pneumonia, chronic bronchitis, bronchiectasis, bronchiolitis, serositis and interstitial lung disease. Most (> 60%) of the patients manifested pulmonary symptoms during periods of quiescent bowel disease, and there was no correlation between age at bowel disease onset, and either the time of onset of respiratory symptoms or the degree of respiratory involvement. Although 8 patients were diagnosed with UC in childhood, all developed respiratory symptoms during adulthood. Moreover, proctocolectomy was not shown to be protective against a recurrence of pulmonary symptoms [1].

In a study by Songur and coworkers, 66% of patients with respiratory symptoms had abnormal pulmonary function tests (PFTs) [10]. More importantly, abnormal PFT's (> 80%), including a reduction in expiratory flow were detected during periods of increased bowel disease activity, as was also noted in pediatric case. In an Eastern European case series, 56% of patients with UC showed a decrease in lung diffusion capacity with no radiographic change. In that study, just 16.7% of these patients were smokers [11]. Although these studies would support the use of PFTs in diagnosing pulmonary disease and following clinical responsiveness to therapy in patients with IBD, the role of routine pulmonary testing has yet to be determined [12]. Longitudinal epidemiological studies may help define the true prevalence of pulmonary disease in children with UC and identify whether risk factors, including family history, smoking, and serological biomarkers can predict this disease phenotype.

## Conclusion

Children with UC may manifest either interstitial or large airway pulmonary involvement. Albeit rare, patients may present with life threatening complications of respiratory disease. Our patient responded to systemic corticosteroid and maintenance anti-metabolite therapy. While there is no epidemiologic pediatric data on the incidence of either infectious or inflammatory pulmonary complications in children with UC, an infectious etiology would still need to be excluded in all patients prior to implementing immuno-modulatory therapy, as was done in our case series. Moreover, all children should be screened for tuberculosis through skin testing, especially now with the increased use of biological therapies.

## Abbreviations

Ulcerative Colitis, UC; Crohn's disease, CD; Interstitial lung disease, ILD; Pulmonary function test, (PFT); Computerized tomography, CT; Erythrocyte sedimentation rate, ESR.

## Competing interests

The author(s) declare that they have no competing interests.

## Authors' contributions

All the authors contributed equally in the patient summary, research, referencing, review, writing and proofreading of the case report.

## Consent

The patient's family had a chance to review the manuscript and provide verbal consent for its submission for publication.
